# The Emerging Role of Mechanics in Synapse Formation and Plasticity

**DOI:** 10.3389/fncel.2018.00483

**Published:** 2018-12-06

**Authors:** Devrim Kilinc

**Affiliations:** INSERM U1167, Institut Pasteur de Lille, Lille, France

**Keywords:** dendritic spine, cytoskeleton, cell adhesion molecules, motor proteins, mechanotransduction, synaptic scaffold proteins

## Abstract

The regulation of synaptic strength forms the basis of learning and memory, and is a key factor in understanding neuropathological processes that lead to cognitive decline and dementia. While the mechanical aspects of neuronal development, particularly during axon growth and guidance, have been extensively studied, relatively little is known about the mechanical aspects of synapse formation and plasticity. It is established that a filamentous actin network with complex spatiotemporal behavior controls the dendritic spine shape and size, which is thought to be crucial for activity-dependent synapse plasticity. Accordingly, a number of actin binding proteins have been identified as regulators of synapse plasticity. On the other hand, a number of cell adhesion molecules (CAMs) are found in synapses, some of which form transsynaptic bonds to align the presynaptic active zone (PAZ) with the postsynaptic density (PSD). Considering that these CAMs are key components of cellular mechanotransduction, two critical questions emerge: (i) are synapses mechanically regulated? and (ii) does disrupting the transsynaptic force balance lead to (or exacerbate) synaptic failure? In this mini review article, I will highlight the mechanical aspects of synaptic structures—focusing mainly on cytoskeletal dynamics and CAMs—and discuss potential mechanoregulation of synapses and its relevance to neurodegenerative diseases.

## Introduction

Chemical synapses of the central nervous system (CNS) mediate the directional information flow between neurons and form the basis of learning and memory. A precisely-defined synaptic cleft separates the opposing pre- and postsynaptic terminals that are held in place via transsynaptic cell adhesion molecules (CAMs). While presynaptic terminals are specialized in neurotransmitter release, postsynaptic terminals house neurotransmitter receptors and various signaling and scaffolding proteins. Functional diversity of synapses is reflected in structural diversity: most inhibitory synapses form directly on the dendrite shaft, while most excitatory synapses form on dendritic spines—morphologically diverse membrane protrusions from the dendrite shaft. Dendritic spines may form enlarged heads with relatively narrow necks, resulting in signaling hubs with restricted electrical and chemical connection to the dendrite shaft. The postsynaptic density (PSD), a dense protein matrix beneath the postsynaptic membrane, forms at the tip of the spine head and orchestrates synaptic functions (Sheng and Kim, 2011). The size and shape of individual spines are regulated in an activity-dependent fashion, supported by a specialized protein synthesis and degradation system (Alvarez-Castelao and Schuman, 2015). Synapses may last from seconds to decades; thus, highly sophisticated regulatory mechanisms are required to effectively control their dynamics, during development and adulthood.

**Figure 1:**
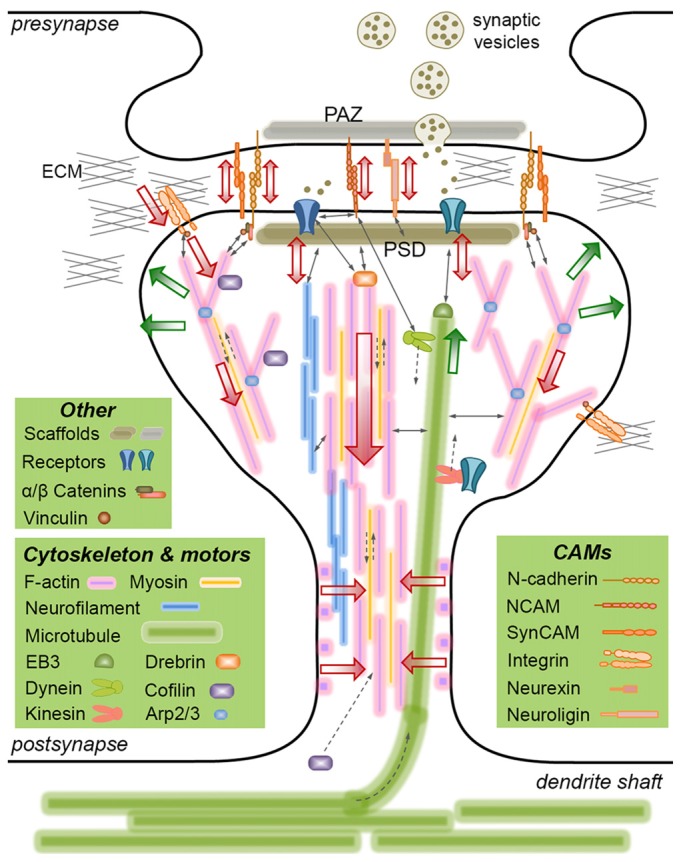
Mechanically-relevant components of an excitatory synapse. Presynaptic vesicle fusion machinery and postsynaptic receptors are held in place by their respective scaffold proteins, which are physically linked to cell adhesion molecules (CAMs) and the cytoskeleton. Direct interactions are indicated with thin, continuous arrows. Translational movements are indicated with thin, broken arrows. Only postsynaptic cytoskeleton is depicted for simplicity. Major cytoskeletal filaments (actin, neurofilaments, microtubules) and a select set of associated molecules (microtubule end-binding protein EB3, actin severing/stabilizing protein cofilin, actin branch-inducing Arp2/3 complex) are depicted. Spine base and center are occupied by stable, dense F-actin, whereas the periphery is occupied by dynamic, branched F-actin. F-actin rings line the spine shaft. Microtubules occasionally invade spines and interact with the postsynaptic density (PSD), but the role of neurofilaments is not clear. Actin and microtubule polymerization creates tensile forces (green arrows) favoring the expansion of the spine head. Myosin motors pull on actin filaments to generate actomyosin contractile forces (red arrows) favoring the shrinkage of the spine head. Kinesin and dynein motors transport cargo on microtubules anterograde and retrograde, respectively. The latter also interacts with the PSD. A select set of CAMs are depicted, most of which form transsynaptic homophilic bonds. N-cadherin and SynCAM bonds encircle the presynaptic active zone (PAZ) and the PSD. β-catenin links N-cadherin to the F-actin cytoskeleton directly or via α-catenin and vinculin. This linkage pulls on transsynaptic CAM bonds, which potentially induce signaling in pre- and postsynaptic compartments, i.e., mechanotransduction. Powered by actomyosin contractile forces, integrins pull on the extracellular matrix (ECM), where force is transmitted via the focal adhesion complex (of which vinculin is a member). The force balance between CAMs and the actin cytoskeleton results in actin retrograde flow and allows for the rapid expansion/shrinkage of the spine structure. Not drawn to scale.

The role of mechanics in neurodevelopment is best characterized at the growth cones (Franze, 2013), highly motile tips of growing axons that integrate mechanical and chemical cues during axon pathfinding (Kerstein et al., 2015). Much less is known about the mechanical aspects of synapse formation and plasticity. This is—partly—due to the increased complexity of mature neurons compared to developing axons. Nevertheless, recent studies established that synapse formation and plasticity require unique mechanisms involving the cytoskeleton, molecular motors, CAMs and the extracellular matrix (ECM; Figure 1). Considering that these components are either force-generating or force-bearing, two critical questions emerge: (i) are synapses mechan(ochem)ically regulated? and (ii) does disrupting the transsynaptic force balance lead to (or exacerbate) synaptic failure? In this mini review article, I will highlight the mechanical aspects of synaptic structures—focusing mainly on cytoskeletal dynamics and CAMs—and discuss potential mechanoregulation of synapses and its relevance to neurodegenerative diseases.

## Dynamic Cytoskeletal Interactions Shape Synapses

The cytoskeleton is an interconnected network of dynamic filaments and regulatory proteins mediating not only the mechanical processes such as shape change and cell motility, but also the global intracellular organization. This is achieved through combining stable, long-range interactions and highly dynamic, short range interactions (Fletcher and Mullins, 2010). Synapses rely on cytoskeletal processes to accomplish specific tasks, from activity-dependent structural change to long-term maintenance of established connections. I will discuss actin, microtubule and neurofilament networks separately, despite the tight coupling between them (Coles and Bradke, 2015).

### Dynamic Actin Networks Control Dendritic Spine Shape and Size

Dendritic spines are structurally supported by a filamentous actin (F-actin) framework, which controls the spine shape and organizes the signaling machinery (reviewed in Hotulainen and Hoogenraad, 2010). The F-actin retrograde flow in spines, i.e., from tip to base, is reminiscent of the same in developing axons (Nichol et al., 2016), albeit with shorter filaments and lower flow rates (~50 nm/s; Frost et al., 2010). However, this resemblance is disputed by reports showing that the F-actin flow slows down (from ~35 nm/s to ~20 nm/s) as dendritic filopodia turn into spines (Chazeau et al., 2014), and that the polarization is lost (Tatavarty et al., 2012). Long-term potentiation (LTP) and depression (LTD) induce actin polymerization and depolymerization in spines causing them to enlarge or shrink, respectively (Bosch and Hayashi, 2012). Three distinct pools of F-actin occupy the dendritic spines: a stable core, forming the center and the base, a dynamic shell, extending towards the periphery, and an apical pool associated with the head enlargement during LTP (Honkura et al., 2008). In addition, there are periodic F-actin rings that shape the spine neck (Bär et al., 2016). The dynamic and stable pools have drastically different turnover rates, i.e., polymer half-life of tens of seconds vs. tens of minutes (Stefen et al., 2016). These differences in F-actin dynamics likely arise from the distinct spatiotemporal organization of various actin-binding proteins (ABPs) in dendritic spines.

Multiple ABPs regulate F-actin in dendritic spines: cortactin and drebrin localize to the stable core, whereas cofilin localizes to the dynamic shell (Rácz and Weinberg, 2013). Cortactin directly interacts with *N*-methyl-*D-aspartate* receptors (NMDARs) and Shank scaffold in the PSD, and regulates the branch-inducing Arp2/3 complex (Hering and Sheng, 2003), which is required for spine maturation (Spence et al., 2016). During LTP, the actin severing protein cofilin is rapidly recruited into the spine and forms stable complexes with F-actin (as cofilin’s effect paradoxically shifts from severing to stabilizing with increasing stoichiometric ratio), which occupy the base of spines and consolidate their expansion (Bosch et al., 2014). Importantly, cofilin regulates NMDA-dependent synapse remodeling in LTP and LTD (Pontrello et al., 2012). Altogether, these data suggest that actin is the primary structural element in postsynapses.

### Microtubules Transiently Invade Dendritic Spines (Not Only) for Cargo Delivery

Microtubules transiently invade mature spines (residing there for a few minutes) and the occurrence and duration of these invasions correlate with neuronal activity (Hu et al., 2008). Transient nature and activity-dependence of microtubule spine invasions suggest that they drive cargo in and out; e.g., kinesin-3 delivers synaptotagmin-IV, an LTP regulator, to spine heads (McVicker et al., 2016). Mitochondria, however, undergo an actomyosin handoff, i.e., switch from microtubule-based to actin-based motor transport, to reach the spine head. Similarly, recycling endosomes containing α-amino-3-hydroxy-5-methyl-4-isoxazolepropionic acid receptors (AMPARs) are transported into spines via myosins Va (Correia et al., 2008) and Vb (Wang et al., 2008). During LTP they undergo syntaxin-4-mediated exocytosis such that AMPAR are inserted into the plasma membrane adjacent to the PSD (Kennedy et al., 2010). However, to what extent AMPAR are actively transported and/or diffuse laterally into the spine head is debated (Penn et al., 2017). Apart from retrogradely transporting neurotrophic factors, dendritic kinesin-4 also regulates microtubule dynamics (Ghiretti et al., 2016), and is required for learning and memory (Muhia et al., 2016).

The microtubule plus end-binding protein EB3 directly interacts with the postsynaptic scaffold protein PSD-95, an event that decreases EB3-microtubule interaction (Sweet et al., 2011), suggesting a functional role for dendritic microtubules in synaptic plasticity. Indeed, spine invasion by EB3-capped microtubules constrains the ABP p140CAP to the PSD and maintains the spine size (Jaworski et al., 2009). Accordingly, during LTD, NMDAR-mediated Ca^2+^ influx removes EB3 from growing microtubule tips, which causes EB3 accumulation in the dendrite shaft and suppresses microtubule entry into the spine (Kapitein et al., 2011). Interestingly, microtubule spine invasions require a cortactin-dependent increase in the F-actin remodeling at the base of the synapse, but do not require EB3- and drebrin-mediated F-actin-microtubule linkages (Schätzle et al., 2018). Altogether, these studies suggest that microtubules indirectly regulate synapses via transporting cargo and regulating the actin cytoskeleton.

### Neurofilaments—Additional Structural Support for Postsynaptic Density?

Neurofilaments are neuron-specific intermediate filaments associated with axon caliber regulation (Lee and Cleveland, 1996). All four neurofilament subunits found in the CNS—NF-L, NF-M, NF-H and α-internexin—localize to the synapses (enriched in postsynapses), are distinct from their axonal counterparts, and have no known functions (Yuan et al., 2015). Neurofilaments—but not F-actin or microtubules—directly interact with SAPAP (Hirao et al., 2000), a member of PSD-95/SAPAP/Shank core complex, the major scaffold of the PSD (Zhu et al., 2017). Mice lacking neurofilament subunits have structurally normal brains, but exhibit synapse plasticity and memory deficits, indicating a functional role. In support of this, dopamine D1 receptor (D1R)-induced LTP was modulated by NF-M, which anchors D1R-containing endosomes to build up a reservoir of D1Rs for their rapid recycling to the postsynaptic membrane (Yuan et al., 2015). Curiously, the cytoplasmic tail of NR1 subunit of NMDAR binds neurofilaments and inhibits their assembly (Ehlers et al., 1998), consistent with the findings that synaptic vesicles and endosomes dock onto neurofilament-based scaffolds, and that vesicle recycling requires neurofilaments to interact with microtubule motors (Yuan et al., 2017). While these data suggest that neurofilaments mainly act as postsynaptic scaffolds, further effort is required to decipher the specific synaptic function(s) of their subunits.

## Cell Adhesion Molecules Initiate, Specify and Regulate Synapses

CAMs are cell surface molecules that link cells to the ECM and to other cells via homophilic and heterophilic interactions. Synaptic CAMs are defined as CAMs with potential to induce synapses via *trans* interactions (Frei and Stoeckli, 2014). However, other CAMs also localize to synapses and contribute to synapse formation and plasticity, through transsynaptic recognition and signaling processes, respectively (Dalva et al., 2007). In fact, the subtype of the CAM(s) recruited can determine the synapse type: for example, alternative splicing of postsynaptic neuroligins leads to either inhibitory or excitatory synapses (Chih et al., 2005). CAMs are hierarchically expressed; some are required for core synaptic functions and others for specialized functions. Moreover, “early” and “late” synaptic genes are co-expressed and co-regulated in the same neuron, suggesting that their differential localization and combinatorial use define synapse specificity and network connectivity rules (Földy et al., 2016). Here, I will describe the major CAM families involved; for a complete list of CAMs in synapses, see recent reviews (Jang et al., 2017; Chamma and Thoumine, 2018).

### Integrins Regulate Functional and Structural Plasticity of Dendritic Spines

Integrins are heterodimeric transmembrane receptors that link ECM components to the actin cytoskeleton via adaptor proteins, e.g., talin and vinculin, forming a “molecular clutch” that transmits actomyosin contractile forces to the ECM (Sun et al., 2016). Integrins regulate the spine functional plasticity through controlling receptor trafficking in a subunit-specific manner: α_3_β_1_-integrins regulate LTP through modulating NMDAR activity (Chan et al., 2007), whereas α_V_β_3_-integrins regulate synaptic strength through stabilizing AMPAR in the membrane (Cingolani et al., 2008). LTP induces long-lasting (~30 min) Rho activity in individual spines that is thought to relay the transient activation of Ca^2+^/calmodulin-dependent protein kinase II (CaMKII; ~10 s to structural plasticity (Murakoshi et al., 2011). Rho and Rac act at different phases of LTP, mediating the spine neck formation and driving the spine head expansion, respectively (Rex et al., 2009). In fact, while ROCK1 regulates the early phase by forming stable actomyosin bundles that create spine polarity, ROCK2 regulates the late phase by controlling Rac activity and by deactivating cofilin (Newell-Litwa et al., 2015). Rac1 activity, in turn, maintains the globular shape of the spine through regulating the localization and dynamics of the branched F-actin network (Chazeau et al., 2014). Separately, integrins regulate the spine structural plasticity through controlling actin remodeling: the integrin/focal adhesion pathway regulates multiple ABPs, including Arp2/3 complex (Serrels et al., 2007), cortactin (Hotulainen and Hoogenraad, 2010) and cofilin (Heredia et al., 2006). Together, these studies suggest that integrins regulate synapses through multiple, intertwined mechanisms. Moreover, recent proteomic analyses identified new, non-canonical adhesion components with emerging functions in mechanotransduction and receptor trafficking (Humphries et al., 2015). Their potential roles in synapse formation and plasticity are yet to be explored.

### Cadherin/Catenin Complexes Are Important Regulators of Synapse Plasticity

Cadherins are Ca^2+^-dependent transmembrane proteins found at intercellular adherent junctions. Their ectodomains form homo- and occasional heterophilic bonds and their intracellular tails interact with various partners—notably catenins and vinculins—to induce downstream signaling. Cadherins can form transsynaptic bonds with different adhesiveness and kinetics, thanks to their large repertoire of homophilic interactions: *trans* dimers (slip bond), X-dimers (catch bond) and clusters, which combine *cis* and *trans* bonds (Leckband and de Rooij, 2014). Cadherin mechanotransduction is highly complex (reviewed in Hoffman and Yap, 2015): on one hand, cadherin-catenin complex directly interacts with F-actin via a two state catch bond (that strengthens under force, as opposed to slip bonds that weaken under force), reinforcing intercellular adhesion (Buckley et al., 2014). On the other hand, force induces a conformational change in α-catenin, revealing cryptic sites for vinculin binding. Activated vinculin not only directly binds to F-actin, but also recruits Ena/VASP family proteins to promote actin assembly, further reinforcing the cadherin-cytoskeleton coupling (le Duc et al., 2010). In the CNS, classical cadherins (e.g., N-cadherin) localize to pre- and postsynapses, and border the presynaptic active zone (PAZ; Uchida et al., 1996). During development, neuron-neuron interactions regulate the activity-dependent dendrite arborization, which is mediated by cadherin/catenin surface levels (Tan et al., 2010). It should be noted that, due to structural differences, N-cadherin and E-cadherin dimers have different disassembly kinetics, where the former depend strongly on Ca^2+^ binding and cannot form X-dimers (Vunnam and Pedigo, 2012). It is therefore likely that N-cadherin and E-cadherin have distinct mechanical behavior. Similarly, αN-catenin differs from αE-catenin in terms of β-catenin binding kinetics (Pokutta et al., 2014), further suggesting that mechanotransduction mechanisms identified for E-cadherin/αE-catenin may not be applicable to synapses.

Cadherins take part in spine and synapse formation, particularly in excitatory neurons (Seong et al., 2015). In postnatal, excitatory synapses, N-cadherin is required for LTP and spine enlargement—but not LTD or spine density and morphology, suggesting that cadherins selectively regulate synapse plasticity (Bozdagi et al., 2010). Indeed, cadherin accumulation on synaptic membranes is required for stabilizing postsynaptic receptors, e.g., kainate receptors (Fièvre et al., 2016) and AMPAR (Mills et al., 2017). Catenins also regulate synapses: upon NMDAR activation, β-catenin is redistributed from the dendrite shaft to the spines (where it binds N-cadherin), leading to synapse enlargement, consistent with its role in learning and memory (Murase et al., 2002). Furthermore, spines compete for surface-bound N-cadherin/β-catenin complexes, which appear to be the key drivers for activity-dependent spine pruning, where β-catenin redistribution determines the fate of individual spines, i.e., stabilizing one while eliminating its neighbors (Bian et al., 2015). Cumulatively, these data suggest that cadherin and its intracellular binding partners are important regulators of synapse plasticity.

### Several Other CAM Families Localize to Synapses

Ectodomains of the immunoglobulin superfamily CAM (IgCAM) contain tandem repeats of Ig-like domains, which permit the formation of a variety of *trans* and *cis* bonds, leading to molecular zippers (Aricescu and Jones, 2007). Such flexibility is ideal for mechanosignaling, where force-induced conformational change regulates intracellular signaling (Johnson et al., 2007). For example, neural CAM (NCAM)—critical for neurodevelopment (Maness and Schachner, 2007)—forms at least two types of homophilic bonds differing in force sensitivity and intercellular distance (Wieland et al., 2005). NCAM regulates synapses by crosslinking to NMDAR and CaMKII via a spectrin-based postsynaptic scaffold (Sytnyk et al., 2006). Additionally, NCAM interacts with dynein to tether microtubule plus-ends, an event that enhances synapse stability (Perlson et al., 2013). SynCAM, originally identified as an IgCAM promoting synaptogenesis (Biederer et al., 2002), forms transsynaptic homophilic bonds at spine heads encircling the synaptic cleft (Perez de Arce et al., 2015). Importantly, SynCAM complexes enlarge during LTD, suggesting that they control the cleft diameter.

Presynaptic neurexins interact with postsynaptic neuroligins, which directly bind to and recruit PSD-95—a function regulated through phosphorylation (Giannone et al., 2013; Bemben et al., 2014). The neurexin-neuroligin bond stabilizes the dendritic filopodia during synaptogenesis (Chen et al., 2010), and regulates the synapse specificity in an isoform-dependent manner (Graf et al., 2004; Boucard et al., 2005). In fact, neurexins are heparan sulfate proteoglycans and neuroligin binding to heparan sulfate chains on neurexin is necessary for synapse development (Zhang et al., 2018). In mature synapses, NMDAR activation leads to juxtamembrane cleavage of neuroligin by matrix metalloproteinase 9 (MMP-9) or by ADAM10, which destabilizes neurexins and decreases synaptic strength by altering presynaptic release (Peixoto et al., 2012; Suzuki et al., 2012).

Another CAM type required for synapse specificity is the clustered protocadherin (Kostadinov and Sanes, 2015). Clustered protocadherins form homodimers and antiparallel homophilic *trans* interactions to regulate dendritic self-avoidance (Nicoludis et al., 2015). Unfortunately, whether CAMs other than integrins and cadherins participate in the mechanotransduction is largely unknown. One exception to this is the ephrin-Eph receptor pair, which bidirectionally regulates synapse formation and maturation in the adult, i.e., ephrins can signal into the Eph-receptor-expressing cell (forward) or into their host cell (reverse; reviewed in Klein, 2009). For example, the dynamics of EphB2 kinase activity at the tip of a dendritic filopodium upon binding to axonal ephrin-B1 determines whether the filopodium retracts or establishes a synapse (Mao et al., 2018). On the other hand, dendritic ephrin-B3 directly interacts with PSD-95 to control its localization and stability, via activity-dependent phosphoregulation (Hruska et al., 2015). Importantly, physically restraining the ephrin-A1 modulates cytoskeletal dynamics by blocking the EphA2 receptor clustering, indicating that ephrin-Eph pair is mechanosensitive (Salaita et al., 2010). Together, these data suggest that mechanotransduction via CAMs may be a general mechanism in synapse regulation, and not unique to integrins and cadherins.

## Other Potential Mechanoregulators of Synapse Plasticity: Scaffold Proteins, Mechanosensitive Ion Channels, and the Extracellular Matrix

Apart from major mechanical actors (cytoskeleton, CAMs), synapses contain other mechanically-relevant components. Synaptic scaffolds support the dynamic components of the PAZ and the PSD (Ziv and Fisher-Lavie, 2014) and, since they physically couple CAMs to the underlying cytoskeleton, they potentially bear tensile forces. While non-specifically pulling on a neurite is sufficient to recruit Bassoon presynaptic scaffold into a potential presynapse (Suarez et al., 2013), the mechanosensitivity of scaffold proteins remains unknown. Mechanosensitive ion channels, however, are expressed in neurons (Hu et al., 2015), although most do not localize to synapses. An important exception to this is NMDAR, which may be activated through increased membrane tension (Paoletti and Ascher, 1994) or by cytoskeletal forces acting on its intracellular domain (Singh et al., 2012). The ECM is another potential mechanoregulator of synapses: the composition, structure and stiffness of the ECM (reciprocally) regulate the cellular mechanotransduction (reviewed in Humphrey et al., 2014). Components of the ECM form a perineuronal net that surrounds dendritic spines and extends into the synaptic cleft (Dansie and Ethell, 2011). In the adult brain, chondroitin sulfate proteoglycans stabilize dendritic spine structure and movement, whereas, other glycoproteins (notably, reelin, agrin and tenascins) are important regulators of synapse plasticity (reviewed in Levy et al., 2014). For example, cleavage of agrin (an integrin α_v_β_1_ ligand) by neurotrypsin promotes LTP by facilitating new dendritic filopodia (Matsumoto-Miyai et al., 2009). Similarly, cleavage of the hyaluronan receptor CD44 by MMP-9, results in its detachment from the ECM and leads to dendritic spine elongation (Bijata et al., 2017). These data suggest that ECM proteolysis regulates synapses, potentially by modifying their force balance.

## Is Synapse Plasticity Mechanically Regulated?

As illustrated above, mechanotransduction takes place during synapse plasticity. It is clear that mechanical processes convey biochemical signals into spine remodeling; however, whether they also convey purely mechanical signals (e.g., forces with a certain magnitude, rate, duration and frequency; Hoffman et al., 2011) to invoke structural change is unknown. In fact, changes in plasma membrane curvature and tension (due to spine remodeling) may be sufficient to induce mechanotransduction (Diz-Muñoz et al., 2013). Identifying such mechanisms remains a challenge due to the high level of complexity, i.e., synapses involve numerous CAMs—some of which crosstalk via common effectors (Mui et al., 2016)—and intertwined cytoskeletal networks (Figure 1). Since pulling on a transsynaptic CAM (e.g., cadherin) bond modulates not only its unbinding rate, but also its downstream signaling by revealing cryptic domains (e.g., α-catenin-vinculin interaction), the acting force needs to be tightly controlled, such that intracellular signaling precedes bond breakage.

Notably, cadherin transsynaptic bonds can be synapse-specific: synapses between hippocampal CA3 and CA1 neurons with high-magnitude LTP require *cis* dimers of postsynaptic cadherins-6 and -10 to form *trans* bonds with presynaptic cadherin-9 (Basu et al., 2017). This level of complexity suggests mechanosignaling to take place, considering the differences in the force-dependency of these bonds. Indeed, N-cadherins stabilize filopodial F-actin through counteracting the actomyosin pulling force—a process associated with the transition of the dendritic filopodia into spines (Chazeau et al., 2015). Remarkably, pulling on N-cadherins at the tips of dendritic filopodia—via optical tweezers—resulted in rapid actin accumulation and mushroom-like spine morphology (Chazeau et al., 2015), suggesting that cadherin mechanotransduction alone can trigger synapse remodeling. Nevertheless, to what degree mechanotransduction and signal transduction mechanisms overlap, whether their activation is synchronized, and whether they operate synergistically (e.g., to strengthen synaptic signaling) remain open questions.

## Is Synapse Mechanics Relevant to Neurodegenerative Diseases?

Whether synapse mechanics is relevant to neurodegenerative diseases is another open question. Synaptic failure is a key event in most neurodegenerative diseases, particularly in Alzheimer’s disease (AD). Accordingly, cytoskeletal proteins, including cofilin (Rahman et al., 2014), drebrin (Gordon-Weeks, 2016) and NF-L (Bacioglu et al., 2016) were implicated in AD pathophysiology. Separately, multiple CAMs, including integrin (Caltagarone et al., 2007), N-cadherin (Andreyeva et al., 2012), NCAM (Leshchyns’ka et al., 2015) and neurexin-neuroligin (Brito-Moreira et al., 2017) are involved in amyloid-β-induced synaptotoxicity, a major event in AD. In fact, amyloid precursor protein (APP), whose cleavage products include amyloid-β, is a transsynaptic CAM (Ludewig and Korte, 2016). APP regulates the PAZ organization (Laßek et al., 2016) and the dendritic spine shape (Weyer et al., 2014), and its dimerization is regulated by N-cadherin (Asada-Utsugi et al., 2011). These observations suggest a link between synapse mechanics and synaptic failure in AD, but direct evidence is missing.

## Conclusions and Outlook

In this mini review article, I attempted to highlight the mechanically-relevant mechanisms that take part in synapse formation and plasticity. Growing evidence suggests that synapses employ mechanosensitive molecules and mechanical processes; however, direct evidence for the mechanoregulation of synapse behavior is currently lacking. Implementing new technologies for force application or measurement (Kilinc et al., 2015), combined with novel single-molecule or super-resolution approaches (Jin et al., 2018) may help discover such mechanisms. To this end, magnetic tweezers would be an excellent tool to specifically-target CAMs on dendritic filopodia (or axon shafts) and to deliver well-defined forces to identify stretch paradigms leading to spine (or PAZ) formation. Downstream effects of the mechanical input may be monitored live in terms of: (i) cytoskeletal dynamics; (ii) activity of cytoskeleton-associated (e.g., ABPs) or signaling proteins (e.g., CaMKII); and (iii) secondary messengers, such as Ca^2+^ or cyclic nucleotides (Blasiak et al., 2017). Notably, the frequency of local Ca^2+^ transients in dendritic filopodia upon initial contact with an excitatory axon determines whether the connection will be lost or stabilized (Lohmann and Bonhoeffer, 2008), suggesting that Ca^2+^ may be an intermediary to mechanical signaling. Finally, single-molecule tension sensors (Cost et al., 2015) may be used to identify load-bearing proteins in synapses during activity-dependent remodeling, as well as to measure forces acting on these proteins as a function of spine size and shape. A better understanding of synapse mechanoregulation could pave the way for mechanically modulated therapies against synaptic failure.

## Author Contributions

DK wrote the manuscript.

## Conflict of Interest Statement

The author declares that the research was conducted in the absence of any commercial or financial relationships that could be construed as a potential conflict of interest.

## Abbreviations

ABP: actin-binding protein
AD: Alzheimer’s disease
AMPAR: α-amino-3-hydroxy-5-methyl-4-isoxazolepropionic acid receptor
APP: amyloid precursor protein
CAM: cell adhesion molecule
CaMKII: Ca^2+^/calmodulin-dependent protein kinase
CNS: central nervous system
D1R: dopamine D1 receptor
ECM: extracellular matrix
F-actin: filamentous actin
IgCAM: immunoglobulin superfamily cell adhesion molecule
LDP: long-term depression
LTP: long-term potentiation
MMP: matrix metalloproteinase
NCAM: neural cell adhesion molecule
NMDAR: *N-methyl-**D-aspartate* receptor
PAZ: presynaptic active zone
PSD: postsynaptic density.
